# Mediating Effect of Filial Piety Between the Elderly’s Family Resource Contribution and Their Family Power: Evidence from China

**DOI:** 10.3389/fpsyg.2022.829678

**Published:** 2022-03-03

**Authors:** Xin Liu, Shuying Bai

**Affiliations:** Department of Humanities, Social Sciences and Law, Harbin Institute of Technology, Harbin, China

**Keywords:** the elderly, filial piety cultural, relative economic income, relative education, intergenerational cohabitation family, family decision-making

## Abstract

With the development of rationalism, although the concept of filial piety is still an important factor affecting family relations, its rules have changed. Based on the resource theory and by measuring family power *via* the role played in family decision-making (FDM), this study explored the mediating role of filial piety norms between elderly’s family resource contributions and family power in intergenerational cohabitation families in Mengzhou city, China. Using a stratified sampling method, 1,200 elderly people were recruited for data collection. Multiple linear regression analysis was used for testing. The results show that (1) the elderly still have some FDM in Chinese intergenerational cohabitation families, and the family power of elderly men is still higher than that of elderly women, which indicate that the influence of traditional patriarchal norms still exists in the family. (2) Filial piety culture mediates between the elderly’s relative economic income (relative to their adult children) and their family power and also mediates the relationship of the elderly’s relative educational level (relative to their adult children) and their family power. It shows that the resources of the elderly relative to their children affect the filial piety of their adult children and then affect the FDM of the elderly. The study is theoretically and practically meaningful.

## Introduction

Since the middle of the 19th century, scholars have formed a common understanding of family development; that is, with the development of industrialization, the family structure has changed from a large family with multiple generations to a small family centered on conjugal relations (Parsons, 1943; Kirby and Smesler, 2012). Intergenerational cohabitation family, however, has become popular again in many developing countries due to the rising housing prices and the need for mutual care or some other reasons (Ruggles and Heggeness, 2008; Zhang et al., 2014; Li and Wu, 2018). Many studies have pointed out that filial piety gradually declined as a result of modernization, which led to the decline of the family status of the elderly (Yan, 2003; Whyte, 2005; Khalaila and Litwin, 2012). The acceleration of the current population aging and the continuous decline of the elderly’s power to participate in family matters constitute a structural tension to some extent. The study on the power status of the elderly and its influencing factors in intergenerational cohabitation families is of great significance not only to the quality of life of an elderly, but also to the stability of his family and, furthermore, to the sustainable development of society.

However, studies on whether the family power of the elderly has risen or not reached an agreement. Filial piety is one of the important factors that affect family status of the elderly, which is also an important part of Chinese traditional family culture (Chen, 2014). It has influenced the parent–child relationship of East Asian peoples for thousands of years (Chow, 1991; Bedford and Yeh, 2019). In traditional societies, the elderly had a high family status which was rooted in the notion of filial piety (Xiaotong, 1985). However, the modernization theory, which was believed as a general rule by many scholars, maintains that family members, especially adult children, were freed from traditional family norms and notions of power as the development of modernization which leads to the diminution of filial piety (Aboderin, 2004; Chow and Bai, 2011). Specifically, in modern society, family property is no longer in the hands of the elderly, and the value of production and life experience owned by the elderly gradually decreases. On the contrary, young people have good skills and channels to obtain information, which makes it easier for them to obtain new ideological resources and to question the value of traditional culture. The authority of the elderly as parents no longer exists and “the resources and power from the parental to the younger generation” (Mason, 1992). As a result, family status of the elderly has declined from the peak in the agricultural society to the lower level in modern industrial society (Cowgill and Holmes, 1972; Vijaya Kumar, 1999; Zuo and Bian, 2005).

However, some scholars doubted that although in the early stage of modernization, the family status of the elderly will gradually decline, with the continuous improvement of the degree of modernization, the elderly will receive more social resources and cultural support, and their family status will be improved again (Palmore and Manton, 1974; Hong and Keith, 1992). Vauclair et al. (2015) who surveyed the elderly in 25 European countries found that in countries with higher degree of modernization, the elderly had better social security and higher employment ratio which enabled them to obtain higher social status and family status. Palmore found that although the status of the elderly declines in many countries, the status of the elderly in Japan is maintained on a high level because the filial piety culture promoted the respect for the elderly (Palmore, 1975).

It can be seen that, under the background of modernization, these scholars put forward different conclusions from different perspectives of resources and filial piety culture. Nevertheless, studies that examine how resource contributions to family and filial piety culture specifically affect the family status of the elderly remain scarce. Therefore, investigating the relationship among filial piety, resource contributions to family, and the family status of the elderly is crucial. In other studies of family relations, such as parental autonomy support and adolescents’ life satisfaction (Tan et al., 2021), caregiving burden and gain among adult–children caring for parents with dementia (Yu et al., 2016), migrant workers’ socioeconomic status and care for their parents diagnosed with terminal cancers (He and Van Heugten, 2020), and so on, filial piety culture is often studied as a mediating variable. Therefore, it is interesting to explore whether or not filial piety culture also plays an intermediary role in the relationship between family resource contribution and family status in modern society.

In addition, the role played in decision-making on family matters is the most direct and fundamental reflection of family power, it also helps to meet the individual needs of the elderly (Safilios-Rothschild, 1976; Zhang and Li, 2019), and therefore, it is reasonable to measure the familial power of the elderly by the role played in family decision-making (FDM).

The organization of this study is as follows: first, based on the resource theory and the review of relevant literature, theoretical framework and hypotheses are made. Then, the survey procedures and measurement methods are presented to test the hypotheses. After the results of hypothesis testing are shown, the main findings of empirical analysis are discussed and research conclusions are drawn. Finally, limitations and directions for future research are given.

## Theoretical Framework and Hypotheses

### Resource Theory

Resource theory is perhaps the most widely used theoretical framework in the study of family power. Although it is based on the relative status of husband and wife in a family, the theory also has the potential to apply in the analysis of the effects of modernization on the relative status of generations (Kung, 2019).

Blood and Wolf put forward the resource theory in the study of marital power. They believed that the internal power of the family depends on the relative material resources that the family members can provide for the family (Blood and Wolfe, 1960). The differences in money, occupation, and other aspects between husband and wife make the relative advantage of the party in the decision-making process. With the development of the resource theory, the concept of resources was extended. Rodman proposed that cultural tradition is also a kind of normative resource (Rodman, 1967). He believed that the distribution of family authority depends on both the relative resources of family members and the family norms prevalent in local society or a specific subculture. In countries and regions where a patriarchal culture prevails, the husband still wields the main power in the family, even if the wife has a higher income and education level than the husband has. However, in the regions with a more prevalent egalitarian culture and higher degree of modernization, the relative resources of the couple play a decisive role in the power relationship between the couples. Family norms define and regulate the meaning and use of relative resources. In short, the resource theory held that material resources and cultural norms are the two main factors that affect the power of husband and wife.

By the same logic, we believe that material resources and cultural norms are also two major factors that affect intergenerational power. In a traditional society of China, traditional norms, such as filial piety, dominate family relationships, but modernization and the development of a market economy have made resources (including income, education, and occupational status) increasingly important factors that affect intergenerational power, which, however, does not mean that the norms of filial piety will no longer have an impact. Specifically, in the transition period of traditional and modern Chinese societies, traditional norms and modernization may play their roles at the same time. Resource theory leads us to consider the roles of resources and filial piety culture on family power. We made some in-depth findings through interviews; that is, there may be an interaction between resources and filial piety culture. Older parents have more resources, which may affect the filial piety of adult children such that adult children have more respect for parents’ family power, which in turn positively affects the elderly’s FDM power. If elderly parents have few resources, it may weaken filial piety of their adult children, which in turn reduces the FDM power of the elderly.

### Resource and Family Decision-Making

Numerous studies have shown that there is a very significant positive relationship between family power (include economic income, educational level, and occupational status) and resources owned by family members (French and Jr Raven, 1957; Zhong, 1989; Inesi et al., 2012). However, due to the uncertainty of occupation, such as the imbalance between occupation and reputation, it is impossible to accurately judge the status of occupation (Kilborune et al., 1994). Specifically, for the elderly who basically quit the labor market, the role of occupation is weak. In addition, previous scholars mostly studied the influence of the absolute resources of the elderly on the intergenerational power, but in fact, the size of resources of the elderly relative to their children is the key to intergenerational power. So, this paper takes education level and personal economic income relative to their adult children as the measurement indices of the resources of the elderly.

Personal economic income is the main source of family material contribution and regarded as an important factor in the operation of family power since economic contribution is explicit and quantifiable and economy is the material basis for family production and life. Some research on couples’ power has shown that the larger the gap between the income of a husband and that of his wife, the more likely the husband is to make more decisions on household matters (Doss, 2013; Zhou and Xiao, 2018). For the elderly, control property has also been considered one critical factor by which they gain or lose status (Williams and Domingo, 1993). The elderly’s relative income level (RIL) with respect to their adult children is closely related to FDM. Economic income of the elderly mainly includes wage income, pension, and so on. The higher economic income an elderly has in an intergenerational cohabitation family, the greater material support he can provide for his family and the greater power he may exercise in the family. On the contrary, if the economic income of an elderly is lower than that of his children, then his income may barely support his own normal life. In such a case, he has to rely on his adult children to live and can make less economic contribution to the family. As a result, the elderly may have less FDM power. Based on the above analysis, the hypothesis is as follows:

H1a: RIL has a positive impact on FDM of the elderly.

On the other hand, the elderly’s education level is also related to their decision-making power. The elderly, with higher levels of education, have stronger rational thinking, and the generation gap between them and their adult children may not be obvious, so they will have more decision-making power in family decisions than less educated seniors (Williams et al., 1999). However, in the past, the measurement was mostly based on the absolute education level of the elderly. As some scholars pointed out, the decision-making power of the elderly in a family is not related to their absolute level, but related to the actual educational differences between parents and their adult children (Williams et al., 1999). Therefore, we measure the education effect by the relative education level (REL) between the elderly and their adult children and propose the following hypothesis:

H1b: REL has a positive impact on FDM of the elderly.

### Resource, Filial Piety, and Family Decision-Making

Filial piety is the core virtue of Confucianism that has influenced the parent–child relationship of peoples in East Asian for thousands of years, and respect and care for the elderly are the two principles of it (Sung, 1995; Funk et al., 2013; Bedford and Yeh, 2019). It plays an important role between resources and family power.

On the one hand, resources affect the filial piety of children. In the traditional society, filial piety is based on the family economy. The elderly mastered the family economic lifeline, so that their children had to respect and obey to them unconditionally (Bedford and Yeh, 2019). However, the development of market economy has broken the traditional family economy. As independent individuals, family members obtain survival resources in the market. The elderly’s proud life experience in the past is no longer respected, since their ability to create economic wealth in society is declining. Children will consider more and more benefit factors when practicing filial piety (Liu and Jia, 2017). The higher the income and education level of the elderly are, the higher the filial piety degree of the children is.

On the other hand, filial piety affects the family power of the elderly. In traditional society, the culture of filial piety is absolutized and religionized. Parent–child is a relationship between leadership and absolute obedience, and the subordinate children also regard obedience to parents as the behavior expected by the society (Sirmon and Hitt, 2010). Filial piety plays a role in maintaining and strengthening the family status and power of the elderly. With the change of society, people have a different understanding of filial piety culture. Some people are prone to the new filial piety advocating equal personalities between parents and children. Although children control the main family resources, they still respect parents’ right of speech. Therefore, they believe that both parents and children should have an equal status in the decision-making process of the family. However, some people believe that if the children control the main material resources of the family and assume the obligation to support the elderly, they should control the main decision-making power, even including the elderly pension decision, which makes some elderly people unable to make the pension decision in line with their expectations (Ying, 2020).

According to the concept of mediating effect (Baron and Kenny, 1986), we believe that filial piety may have a mediating effect between the resources and family power of the elderly.

In addition, respect for the elderly is one of the most important aspects of filial piety culture, both in traditional society and in modern society of China. In the traditional society of China, the essence of filial piety was an ideology of respecting the elderly. The Analects of Confucius emphasizes the importance of respecting the elderly. Tsze-yu asked what filial piety was. Confucius replied, “The filial piety nowadays means the support of one’s parents. But dogs and horses likewise are able to do something in the way of support, without reverence, what is there to distinguish the one support given from the other?” (Li, 1967). In the context of social transformation and population aging in China, respect for the elderly is still an important manifestation of filial piety culture (Cao and Hu, 2021). In some other Asian countries, respect for the elderly is also a major measure of filial piety (Palmore, 1975). In addition, the measurement of filial piety in the previous literature is always based on the children’s attitude, such as “I think I have the responsibility and obligation to take care of my parents,” or “I never speak loudly to my parents, even when I am angry or irritated” (Kao and Travis, 2006; Khalaia, 2010). However, to determine whether children are filial or not should also take into account the feelings of the elderly, that is, whether the elderly think their children are filial. Therefore, this study measures filial piety by asking the elderly how much they felt being respected by their children (RAC) in the family and proposes the hypotheses:

H2a: RAC positively mediates the relationship between RIL and FDM of the elderly.

H2b: RAC positively mediates the relationship between REL and FDM of the elderly.

**FIGURE 1:**
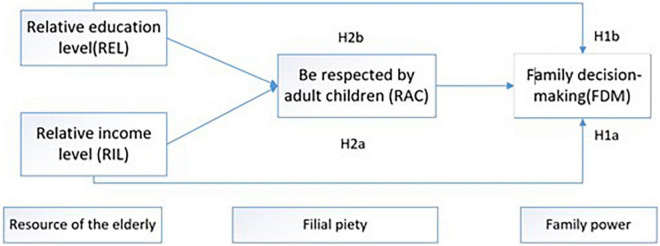
Theoretical model.

The theoretical model of this study is summarized in Figure 1.

## Data Processing and Analysis Method

### Survey Procedure

The data were collected in Mengzhou city, Henan Province of China. Henan Province is located in the center of China, the true “heart of China.” As one of the most important birthplace of Chinese civilization and Chinese nation, Henan Province is profound of cultural heritage. Mengzhou city, located in the northwest of Henan Province, is not a highly modern city like Shanghai. On the one hand, people living there are affected by modernization and network culture, and their values are diversified. On the other hand, traditional family culture still plays an important role in their life. Therefore, the investigation of intergenerational cohabitation families in Mengzhou city can better reflect what changes have taken place in the resources, people’s family role cognition and family intergenerational power under the alternating influence of traditional culture and modernization with the rapid development of China’s economy.

We designed the questionnaire in the following steps. First, we conducted in-depth interviews with 10 elderly people in different ages and obtained a better understanding of their family power. Second, based on these interviews and the measurement scales from references, we designed a questionnaire in English and then translated the questionnaire into Chinese by three Ph.D. students fluent in both languages. To avoid cultural bias and to ensure accuracy, the Chinese version was then back-translated into English by two bilingual translators and the back-translation was compared with the original English version. Third, a pilot test was conducted among 50 Chinese elderly people in different ages. Based on their feedback, the questionnaire was further improved and finalized.

Stratified random sampling method was used to select samples in Mengzhou city of China. First, we randomly selected 2–3 streets from each subdistrict offices and two villages from each town or countryside^1^. Second 2–3 communities were randomly selected from each street, and three village groups (the lowest administrative unit in Chinese villages) were randomly selected from each village. Finally, corresponding samples of the elderly (≥60 years old) were selected. The simple random sampling was conducted within each stratum. The sampling distribution is shown in Table 1.

**TABLE 1:** Sampling distribution.

Unit name	Population over 60 years old	Sample size
DD subdistrict	7872	120
HC subdistrict	9685	147
HY1 subdistrict	3811	58
HY2 subdistrict	6392	97
The town of HG	7385	112
The town of HZ	8577	130
The town of CB	7645	116
The town of GD	6539	99
The town of ZH	8618	131
The town of XG	7084	108
The countryside of HS	5397	82
The city of M (total)	79005	1200

After that, we recruited three Ph.D. students and trained them before the interview. Then, we asked them to present questionnaires to the elderly in intergenerational cohabitation families. The data collection process lasted for 9 months with all the 1,200 questionnaires distributed. After eliminating invalid questionnaires with serious random answers and/or missing answers, we finally collected 1,021 valid questionnaires. The effective response rate was 85.1%.

This study conforms to the guidelines of Declaration of Helsinki and was approved by Ethics Committee of Harbin Institute of Technology, China. All participants voluntarily answered the assessment tools after their formed consent was obtained. The Ph.D. students involved in the data collection have also been professionally trained and signed responsibility letters before the investigation.

### Measurement

#### Dependent Variables

In the past, few studies were conducted on the decision-making power in intergenerational families, compared with studies focusing on the measurement of the decision-making power of husband and wife.

To comprehensively and objectively measure the FDM power of intergenerational cohabitation elderly, we referred to the previous literature on the measurement dimension of husband and wife’s FDM power (Blood and Wolfe, 1960; Dan, 2004) and combined with the research results of intergenerational cohabitation families and then constructed a FDM scale including seven indicators: daily expenditure, life insurance purchase, investment or loan, housing choice, medical treatment, adult children’s career choice, and grandchildren’s living arrangements. The seven indicators were all measured by five-point Likert’s scoring method, with 1 corresponding to “unable to make decisions,” and 5 to “completely able to make decisions.” The higher the score is, the greater decision-making power the elderly have at home.

We performed reliability and validity analyses on FDM. Factor analysis was used to measure the construct validity of the FDM. The result showed that KMO (Kaiser–Meyer–Olkin) was 0.873, higher than 0.7. This means that the data were suitable for performing factor analysis. The results of Bartlett spherical test were also significant, which means that there was a significant correlation between variables (*p* < 0.001). These showed that there was sampling adequacy. The average variance extracted (AVE) for FDM was above 0.5. The composite reliability (CR) surpassed the recommended the cutoff of 0.7. The factor loading of each measurement item was highly significant (*p* < 0.001), ranging from 0.670 to 0.845, which indicates the unidimensionality of the measures. The respective Cronbach’s alpha value of FDM was 0.880, higher than 0.7, which indicates that the data have a high reliability. The results are shown in Table 2.

**TABLE 2:** Validity and reliability assessment of FDM.

Variables	Measurement Items	Factor Loadings	AVE	CR	Cronbach’s alpha
FDM	Daily expenditure	0.845	0.585	0.908	0.880
	Life insurance purchase	0.727			
	Investment or loan	0.830			
	Housing choice	0.811			
	Medical treatment	0.763			
	Adult children’s career choice	0.670			
	Grandchildren’s living arrangements	0.691			

*AVE, average variance extracted; CR, composite reliability.*

#### Explanatory Variables

Explanatory variables can be divided into independent variables and intermediary variables. Independent variables include REL and RIL. RIL is measured by the question “Is your income level higher than or lower than your adult children’s (if living with multiple adult children, then compared with the highest income among adult children)?”. We used five-point Likert-style scoring method to indicate the income gap from “far lower than my adult children” to “far higher than my adult children.” The higher the score is, the more the income of the elderly is higher than that of the adult children. REL is measured by the question “Is your education level higher than or lower than your adult children’s (if living with multiple adult children, then compared with the highest educational level among adult children).” It also used five-point Likert-style scoring method to indicate the educational level gap from “far lower than my adult children” to “far higher than my adult children.” The higher the score is, the more the educational level of the elderly is higher than that of the adult children. Intermediary variable is filial piety which is measured by the question “How much do you feel respected by your adult children?” It was also measured by the five-point Likert scale, the higher the score, the more respected the respondent felt by their adult children.

In addition, we selected three demographic variables that might affect the statistical results as control variables including age, ability of daily living (ADL), and residence (city or country), which may cause the change of dependent variables.

The basic characteristics of each variable are shown in Table 3.

**TABLE 3:** The basic characteristics of each variable.

Variables	Frequency (Relative frequency)					Mean
	1	2	3	4	5	
**Dependent variable**						
Daily expenditure	65 (6.4%)	335 (32.8%)	126 (12.3%)	282 (27.6%)	213 (20.9%)	3.24
Life insurance purchase	77 (7.5%)	212 (20.8%)	446 (43.7%)	178 (17.4%)	108 (10.6%)	3.03
Investment or loan	334 (32.7%)	208 (20.4%)	337 (33%)	78 (7.6%)	64 (6.3%)	2.34
Housing choice	109 (10.7%)	194 (19%)	485 (47.5%)	142 (13.9%)	91 (8.9%)	2.91
Medical treatment	66 (6.5%)	156 (15.3%)	510 (50.0%)	158 (15.5%)	131 (12.8%)	3.13
Adult children’s career choice	376 (36.8%)	313 (30.7%)	253 (24.8%)	57 (5.6%)	22 (2.2%)	2.06
Grandchildren’s living arrangements	287 (28.1%)	337 (33.0%)	271 (26.5%)	83 (8.1%)	43 (4.2%)	2.27
**Independent variables**						
RIL	532 (52.1%)	252 (24.7%)	150 (14.7%)	68 (6.7%)	18 (1.8%)	1.81
REL	70 (6.9%)	542 (53.1%)	302 (29.6%)	106 (10.4%)	1 (0.1%)	2.44
**Intervening variable**						
RAC	18 (1.8%)	117 (11.5%)	323 (31.6%)	349 (34.2%)	214 (21.0%)	3.53

### Analysis Method

Mediating effect was investigated mainly with multiple stepwise regression analysis by software SPSS 26.0. According to R. Baron and D. A. Kenny, the premise of mediating effect is the correlation between variables (Baron and Kenny, 1986). Therefore, we first conducted a correlation analysis on the variables of housing ownership, intergenerational income gap, role cognition of the elderly and adult children, and FDM power (common factor 1) and then used the multiple stepwise regression analysis method to test the hypotheses. Stepwise analysis is the most commonly used method to test the mediating effect. By constructing three models, the regression coefficient was tested to determine whether the mediating effect is significant (Judd and Kenny, 1981; Wen et al., 2004).


$$\left(\mathrm{Model}\,1\right)\,\text{Y}=\beta_{1}\,\mathrm{RIL}+e_{1}$$

$$\left(\mathrm{Model}\,2\right)\,M=\beta_{2}\,\mathrm{RIL}+e_{2}$$

$$\left(\mathrm{Model}\,3\right)\,Y=\beta_{3}\,\mathrm{RIL}+\beta_{4}\,M+e_{3}$$

The first step is to test the correlation between independent and dependent variables. This is the basis for testing whether the mediating effect is significant. Model 1 is the relationship model between independent and dependent variables. In Model 1, Y represents FDM of the elderly, and the coefficient β_1_ represents the degree of influence of RIL on FDM. If the coefficient β_1_ is significant, namely H0: β_1_ = 0 is rejected, which indicates that RIL has a significant influence on FDM.

The second step is to test the correlation between independent and intermediary variables (Model 2), where M is an intermediary variable representing RAC. If the coefficient β_2_ is significant, namely H0: β_2_ = 0 is rejected, which indicates that RIL of the elderly has a significant influence on RAC, and the mediating effect is significant.

The third step is to test the total effect. When the above two conditions are satisfied, in Model 3, it will be called complete mediation if coefficient β_3_ is not significant and RIL will partially mediate between RIL and FDM if β_3_ and β_4_ are significant.

The same steps can be followed to test the mediating effect of RAC between REL and FDM.

## Results

Table 4 shows the correlation coefficient results among all the analysis variables. It can be seen that there is a significant positive correlation between REL, RIL, RAC, and FDM of the elderly in intergenerational cohabitation family. At the same time, there is a very significant positive relationship between REL and RAC, RIL and RAC, and RAC and FDM. It is worth noting that there are significant gender differences in FDM and RAC of the elderly in intergenerational cohabitation families. FDM and RAC of the elderly men are still significantly higher than those of the elderly women. It shows that the influence of patriarchal culture still exists in this region. In addition, there are significant differences in the FDM power of the urban and rural elderly, and the FDM power of the urban elderly is higher than that of the rural elderly. It also can be seen that the research variables in the theoretical model shown in Figure 1 are significantly correlated with each other, which can be used to further analyze the mediating effect of filial piety culture.

**TABLE 4:** The correlation matrix between variables.

Variables	1	2	3	4	5	6	7	8
(1) ADL	1	−0.332***	–0.058	0.136**	0.223***	–0.046	0.174***	0.054
(2) Age	−0.332***	1	0.066*	0.018	−0.315***	–0.037	−0.149***	–0.030
(3) Male(female)	–0.058	0.066*	1	0.103**	0.062*	0.170***	0.090**	0.091**
(4) City(countryside)	0.136**	0.018	0.103	1	0.073*	0.056	0.348***	0.294***
(5) FDM	0.223***	−0.315***	0.062*	0.073*	1	0.220***	0.398***	0.250***
(6) REL	–0.046	–0.037	0.170***	0.056	0.220***	1	0.172***	0.135***
(7) RIL	0.174***	−0.149***	0.090**	0.348***	0.398***	0.172***	1	0.214**
(8) RAC	0.054	–0.030	0.091**	0.294***	0.250***	0.135***	0.214**	1

*p < 0.05, **p < 0.01, ***p < 0.001.

Hierarchical regression analysis was used to further verify our hypotheses, and the results are shown in Table 5. We used multiple linear regression equation to test the main effect and intermediary effect of each independent variable. To test the relationship among REL, RIL, and FDM, Models 1 and 2 were constructed with FDM as the dependent variable. Model 1 includes four control variables: age, gender and ADL, and two more variables are added to Model 2, that is, RIL and REL. Compared with Model 1, the adjusted *R*^2^ in Model 2 is significantly higher. It means that the explanatory authority of the model is enhanced. Specifically, compared with their adult children, the elderly with higher income have greater decision-making power (β = 0.340, *p* < 0.001). As a result, H1a is verified. Compared with their adult children, the elderly with higher education level have greater decision-making power (β = 0.157, *p* < 0.001), and H1b is verified.

**TABLE 5:** Mediation regression models.

Variable	FDM						RAC	
	Model 1	Model 2	Model 3	Model 4	Model 5	Model 6	Model 7	Model 8
ADL	0.128***	0.108***	0.095**	0.096**	0.142***	0.139***	–0.005	0.013
City(countryside)	0.052	–0.069	–0.069	−0.117***	0.041	–0.018	0.247***	0.281***
Age	−0.279***	−0.224***	−0.231***	−0.227***	−0.264***	−0.258***	–0.022	–0.030
Male(female)	0.083**	0.033	0.057*	0.046	0.048	0.039	0.056	0.046
RIL		0.340***	0.366***	0.343***			0.121***	
REL		0.157***			0.206***	0.183***		0.110***
RAC				0.194***		0.211***		
Adjusted *R*^2^	0.122	0.257	0.235	0.268	0.162	0.202	0.100	0.099
F	36.363***	59.870***	63.590***	63.260***	40.494***	43.951***	23.556***	23.399***

*p < 0.05, **p < 0.01, ***p < 0.001.

To test the mediating effect of RAC, four models (Models 3 through 8) were constructed. Models 3 and 4 were used to test the influence of RIL effect on FDM before and after RAC was added as an independent variable; Models 5 and 6 were used to test the influence of REL effect on FDM before and after RAC was added as independent variable; Models 7 and 8 were used to test the influence of RIL and REL effects on RAC. From the regression results of each model:

(1) Models 7 shows that RIL is related to RAC, β = 0.121, *p* < 0.001; Model 8 shows that REL is related to RAC (β = 0.110, *p* < 0.001).
(2) Models 3 and 4 show that with the RAC added in the model, the effect coefficient of RIL effect on FDM of the elderly decreases from 0.366 to 0.343, which indicates that RAC plays a partial mediating role between RIL and FDM of the elderly living with their adult children. H2a is verified.
(3) Models 5 and 6 show that the coefficient of REL effect on FDM decreases from 0.206 to 0.183 with RAC added to the model, which indicates that RAC also plays a partial mediating role between REL and FDM of the elderly living with their adult children. H2b is verified.

## Conclusion

### Main Findings

Living with their children can ensure the quality of life of the elderly to a certain extent, but the decline of family power of the elderly may reduce the enthusiasm and subjectivity of their lives. Therefore, it is of great significance to explore the family power and influence mechanism of the elderly living intergenerationally. However, previous studies ignored the mediating role of filial piety culture. Filial piety culture is an important part of Chinese traditional family culture, which is still of great significance to family stability and social sustainable development in modern society. Based on the resource theory, this paper explores the influencing mechanism of family power from the elderly resources (relative to their adult children) and filial piety culture, specifically examines the relationship between the elderly resources and family power, and focuses on verifying the mediating role of filial piety culture. The analysis results confirm the research hypothesis.

First, from the perspective of intergenerational power distribution, the elderly still have some decision-making power in family matters in Chinese intergenerational cohabitation families, contrary to some prediction opinions of family modernization theory (Palmore and Manton, 1974; Whyte, 2005; Chow and Bai, 2011). The reason may lie on the resources the elderly contributed to their family. With the rapid development of society, although the income of many Chinese adult children exceeds that of their parents, the rising housing price and living cost in China make adult children’s life more and more stressful. This fact may explain why more and more adult children in China prefer to live with their elderly parents in the trend of family centralization and individualization. If the elderly parents can provide their children with housing and some financial help, it will greatly relieve the living pressure of adult children. The elderly with better material capital transfer their material resources to their adult children to ensure the family’s material life quality. This fact not only supports to the reduction of adult children’s life pressure, but also is conducive to elderly’s intervention and control of adult children’s life. This also reflects that in modern society of China, the emotional neutrality of family relations is obvious. The distribution of intergenerational power is no longer determined by the traditional Chinese hierarchy of elderly and children, but in such a way that the elderly obtain certain family power by transferring resources to their adult children. This finding, from the quantitative perspective, also verifies some previous research conclusions drawn through qualitative analysis on Chinese families (Zhang and Li, 2019; Yuan, 2021). In addition, older men have more FDM power than older women. In the family intergenerational power distribution, the family power of elderly men is still higher than that of elderly women, which indicates that the influence of traditional patriarchal norms still exists in the family. This is consistent with some previous research which suggests that older women may still be disadvantaged in the family (Lee and Hong-kin, 2005; Kumar and Bhakat, 2020).

Second, the filial piety cultural norms in the family play a partial intermediary role between resources and family power of the elderly. The degree of respect of the elderly by their children is still important in the FDM process. On the one hand, the degree of respect for the elderly by their children is positively correlated with their own resources. Compared with the elderly with poor material and cultural resources, the elderly with rich material and cultural resources are more respected by their children. The process of modernization is also the process of interpersonal relationship remodeling. Even the intergenerational relationship constructed by blood ties, the tendency of rationalism and utilitarianism is also obvious. The degree of respect for the elderly by children is no longer only dependent on the traditional family norms of elderly and children, but is also significantly affected by the amount of material, cultural, and other resources owned by the elderly. Some scholars have found that in modern society, filial piety is reciprocal, which emphasizes children’s care and respect to their parents (Fan, 2014). Our study, however, shows that filial piety in modern society is reciprocal based on rationalism. Adult children’s respect for their older parents is inseparable from their parents’ resources. On the other hand, the process of modernization is also the process of interpersonal relationship remodeling. The cultural norms of family’s filial piety will positively affect their family power. The higher the degree of respect for the elderly by their children is, the greater the family power will be. This also shows that no matter it is “authoritative filial piety” or “reciprocal filial piety,” it still has a certain guarantee for the power status of the elderly at home, as long as the connotation of family filial piety contains the behavioral norms of respect for the elderly by their children.

### Theoretical Contributions and Practical Implications

This study contributes to the literature in several ways.

First of all, the researches on the family power of the elderly were mostly from a macroperspective, that was, the impact of modern social changes on the status of family power of the elderly (Cowgill and Holmes, 1972). Even if some studies were based on a resource and cultural perspective which were measured by the impact of macropolicies and cultural differences resulting from different levels of modernization and was still based on a macro perspective, however, it is reasonable to view the family power of the elderly from a microperspective, and the resources and culture within the family are the main factors that affect the family power of the elderly. Therefore, this study, based on the resource theory of husband and wife’s power, deeply explores the influencing mechanism of the family power of the elderly and makes contributions to enriching the theory of the family power of the elderly.

Second, we applied the resource theory used in the study of conjugal power to the intergenerational power, which enriches the theory of intergenerational power. What is more, although the resource theory is based on the study of conjugal power mentions where resources and patriarchal culture may have an impact, previous studies mostly examined patriarchal culture through regional comparisons, that are, compared with families in regions with different degrees of modernization, what are the differences in family power of family members (Rodman, 1967). There is no in-depth exploration of the role of culture in the influence mechanism of family power. For the elderly, filial piety is the main culture factor that affects their family power, and filial piety culture is also a subjective concept and attitude of people. The resource contribution of the elderly to the family will affect the filial piety concept of family members and the attitude toward the elderly, especially in the modern society where rationalism is prevalent. Through our investigation, it is confirmed that there is a mediating effect of filial piety culture in the influence mechanism of family power.

With the aggravation of the aging problem in many countries including China, many scholars put forward the view of active aging and found that with the extension of life expectancy, the elderly can still play an important role in their family and society, such as taking care of grandchildren in the family (Jessica et al., 2018) or providing support for non-kin in the society (Pelle et al., 2021). In addition, with better resources, they can have more material foundation and social network foundation to make more contributions to the family and society and also affect the filial attitude of their children and their family power. Based on this, we put forward the following two practical implications: on the one hand, it is necessary to give full play to the economic and spiritual potential of the elderly and to improve the social participation rate of the elderly. The elderly can actively participate in economic, social, cultural, and political life through the elderly university and reemployment, so that the elderly can be useful, thereby improving the quality of life of the elderly and promoting the harmony of family relations; on the other hand, more attention should be paid to family culture, especially the role of family “filial piety” culture in the core values of socialism in the new era. In the construction of family style, we should advocate the harmonious atmosphere of respecting and loving the elderly, carry forward the culture of respecting and respecting the elderly, transform it into the emotional identity and behavior habits of the younger generation, and meet the rights needs of the elderly, so that the elderly can truly enjoy their old ages, and also, the transformation of the cultural atmosphere within the family can be promoted to a more equal, fair, and civilized direction.

### Limitations

Based on the survey data of intergenerational cohabitation elderly in Mengzhou city, Henan Province of China, this paper makes an empirical analysis and draws some enlightening research conclusions, but our research can be further improved. First, this paper focuses on the family power of urban and rural intergenerational cohabitation elderly in central China, and the differences in regional characteristics may also affect the relationship between variables. Future research can also conduct a more comprehensive analysis by comparing the survey data of different regions. Second, this study has a horizontal nature which cannot reflect the longitudinal time evolution of family power of the elderly in intergenerational cohabitation families. In the future, longitudinal time comparative analysis can be carried out to analyze how the relationship among resources, filial piety culture, and family power of the elderly change over time.

## Data Availability Statement

The raw data supporting the conclusions of this article will be made available by the authors, without undue reservation.

## Ethics Statement

The studies involving human participants were reviewed and approved by Ethics Committee of Harbin Institute of Technology, China. The patients/participants provided their written informed consent to participate in this study.

## Author Contributions

Both authors listed have made a substantial, direct, and intellectual contribution to the work and approved it for publication.

## Conflict of Interest

The authors declare that the research was conducted in the absence of any commercial or financial relationships that could be construed as a potential conflict of interest.

## Publisher’s Note

All claims expressed in this article are solely those of the authors and do not necessarily represent those of their affiliated organizations, or those of the publisher, the editors and the reviewers. Any product that may be evaluated in this article, or claim that may be made by its manufacturer, is not guaranteed or endorsed by the publisher.

## Footnotes

1 In the hierarchical administration architecture of a city of China, countryside and town, subordinate to the city, are at the same administrative level, each of which consists of several villages.
